# Advances in Targeting Signal Transduction Pathways

**DOI:** 10.18632/oncotarget.802

**Published:** 2012-12-30

**Authors:** James A. McCubrey, Linda S. Steelman, William H. Chappell, Lin Sun, Nicole M. Davis, Stephen L. Abrams, Richard A. Franklin, Lucio Cocco, Camilla Evangelisti, Francesca Chiarini, Alberto M. Martelli, Massimo Libra, Saverio Candido, Giovanni Ligresti, Grazia Malaponte, Maria C. Mazzarino, Paolo Fagone, Marco Donia, Ferdinando Nicoletti, Jerry Polesel, Renato Talamini, Jörg Bäsecke, Sanja Mijatovic, Danijela Maksimovic-Ivanic, Michele Milella, Agostino Tafuri, Joanna Dulińska-Litewka, Piotr Laidler, Antonio B. D'Assoro, Lyudmyla Drobot, Kazuo Umezawa, Giuseppe Montalto, Melchiorre Cervello, Zoya N. Demidenko

**Affiliations:** ^1^ Department of Microbiology and Immunology, Brody School of Medicine at East Carolina University; ^2^ Department of Communication Sciences and Disorders, College of Allied Health Sciences, East Carolina University, Greenville, North Carolina, USA; ^3^ Dipartimento di Scienze Biomediche e Neuromotorie, Università di Bologna, Bologna, Italy; ^4^ Institute of Molecular Genetics, National Research Council-IOR, Bologna, Italy; ^5^ Department of Bio-Medical Sciences, University of Catania, Catania, Italy; ^6^ Unit of Epidemiology and Biostatistics, Centro di Riferimento Oncologico, IRCCS, Aviano, Italy; ^7^ Department of Medicine, University of Göttingen, Göttingen, Germany; ^8^ Department of Immunology, Instititue for Biological Research “Sinisa Stankovic”, University of Belgrade, Belgrade, Serbia; ^9^ Regina Elena National Cancer Institute, Rome, Italy; ^10^ Sapienza, University of Rome, Department of Cellular Biotechnology and Hematology, Rome, Italy; ^11^ Chair of Medical Biochemistry, Jagiellonian University Medical College, Kraków, Poland; ^12^ Department of Biochemistry and Molecular Biology, Mayo Clinic College of Medicine, Rochester, MN, USA; ^13^ Palladin Institute of Biochemistry, National Academy of Sciences of Ukraine, Kyiv, Ukraine; ^14^ Department of Molecular Target Medicine Screening, Aichi Medical University School of Medicine, Nagakute, Aichi, Japan; ^15^ Biomedical Department of Internal Medicine and Specialties, University of Palermo, Palermo, Italy; ^16^ Consiglio Nazionale delle Ricerche, Istituto di Biomedicina e Immunologia Molecolare “Alberto Monroy”, Palermo, Italy; ^17^ Department of Cell Stress Biology, Roswell Park Cancer Institute, Buffalo, New York, USA

**Keywords:** Targeted Therapy, Therapy Resistance, Cancer Stem Cells, Raf, Akt, PI3K, mTOR, AMPK, Metformin

## Abstract

Over the past few years, significant advances have occurred in both our understanding of the complexity of signal transduction pathways as well as the isolation of specific inhibitors which target key components in those pathways. Furthermore critical information is being accrued regarding how genetic mutations can affect the sensitivity of various types of patients to targeted therapy. Finally, genetic mechanisms responsible for the development of resistance after targeted therapy are being discovered which may allow the creation of alternative therapies to overcome resistance. This review will discuss some of the highlights over the past few years on the roles of key signaling pathways in various diseases, the targeting of signal transduction pathways and the genetic mechanisms governing sensitivity and resistance to targeted therapies.

## Mutations Alter the Activity of the Ras/Raf/MEK/ERK and PI3K/PTEN/Akt/mTOR pathways

The Ras/Raf/MEK/ERK and PI3K/PTEN/Akt/mTOR pathways are often activated by mutations within individual components of these pathways, as well as the aberrant activation of upstream growth factor receptors. The genetic basis of sensitivity and resistance to various small molecule inhibitors which target these pathways as well a comprehensive list of small molecule inhibitors as a well as their use in clinical trials have recently been published [1-7].

## Predicting Sensitivity to Small Molecule Inhibitors

Extensive panels of human cell lines have been examined for mutations in genes implicated in cancer as well as for their sensitivity to various inhibitors and chemotherapeutic drugs commonly used to treat cancers [8,9].

The cell lines were intensively interrogated by expression profiling, chromosome copy number, deep sequencing, biostatistical and systems analyses. Both studies indicated that sensitivity to inhibitors was often linked with genetic mutations at key elements in the Ras/Raf/MEK/ERK, PI3K/PTEN/Akt/mTOR and some other pathways. Sensitivity to MEK and Raf inhibitors was often investigated in these studies. Sensitivity to the B-Raf inhibitor PLX4720 was highly associated with particular mutations at *BRAF* (V600E). Sensitivity to MEK inhibitors was shown to be associated with *BRAF*, *NRAS* as well as *PTEN*, *PTPN5*, *SPRY2*, *DUSP4*, *DUSP6* mutations and to a lesser extent mutations at *KRAS*. Sensitivity to MEK inhibitors in *NRAS* mutant lines was linked with aryl hydrocarbon receptor (*AHR*) expression [9].

Mutation of *BRAF* or *RAS* can contribute to the pathogenesis of many cancers, including, melanoma and colo-rectal cancer [10]. *BRAF* mutations have also been implicated in the pathogenesis of papillary thyroid cancer [11].

Mutation of *BRAF* and *KRAS* can result in aberrant c-Myc and SIRT1 protein deacetylase expression in the colorectal cancer [12]. Mutations at *KRAS* which result in increased mutant Ras activity can interact with increased Wnt expression in lung cancer and lead to a worse prognosis as the tumors arise at an increased incidence and tumors which are also larger [13]. Interesting it was observed that in those tumors where there was increased KRAS and Wnt activities they had a distinct phenotype which was similar to embryonic progenitors found in the developing lung, consistent with the previously described effects of the Wnt pathway on developmental processes.

## Novel Targets Interacting with the Ras/Raf/MEK/ERK Pathway

Novel upstream Shc regulators such as the multiple copies in T-cell malignancy 1 (*MCT-1*) gene have been recently described which may become an important therapeutic target as MCT-1 is co-activated with Shc gene in human carcinomas and knockdown of MCT-1 enhances apoptotic cell death [14].

The CDC28 protein kinase regulatory subunit 1B (*CKS1B*) gene is involved in multiple myeloma (MM). There is an inverse correlation between CKS1B expression and survival and it may be a novel therapeutic target [15]. Recently it was shown that CKS1B activates both the Raf/MEK/ERK and STAT3 pathways and promotes drug resistance [16]. Targeting CKS1B may be a novel approach to treat certain cancers.

## Advances in Our Understanding and Targeting of the Ras/Raf/MEK/ERK and PI3K/PTEN/mTOR Pathways

Effective inhibitors specific for many of the key components of the Ras/Raf/MEK/ERK/MNK, Ras/PI3K/PTEN/mTOR and other pathways have been developed. These pathways are often implicated in therapeutic resistance and interact with many pathways. [17-23].

Ras inhibitors have been examined in many studies including clinical trials. However the results so far have not been encouraging. Salirasib (Farnesythiosalycilic acid, FTS) is a Ras inhibitor. It has recently been demonstrated that FTS treatment of immuno-competent mice with subcutaneous or intracranial brain tumors resulted in a favorable antitumor environment [24]. The authors observed an increase in regulatory T-cells which altered the tumor microenviroment and preventing the resistance of the tumor cells to destruction by the immune system.

BRAF inhibitors have been shown to be effective in the treatment of melanomas. One of the first described Raf inhibitors, Sorafenib, has been shown to inhibit many targets, including VEGFR, PDGFR and Raf. It is approved for treatment of hepatocellular carcinoma (HCC) where there are few effective therapeutic approaches [25]. The mechanism of actions of sorafenib have been further elucidated and it has been determined to alter the expression of many genes in HCC [26]. A recent genomic analysis indicated that sorafenib significantly altered expression levels of 826 and 2011 transcripts in HepG2 and Huh7 cells, respectively. The affected genes whose expression were upregulated in response to sorafenib treatment were determined to be involved in angiogenesis, apoptosis, transcription regulation, signal transduction, protein biosynthesis and modification, In contrast, genes which were determined to be downregulated after sorafenib treatment were involved in cell cycle control, DNA replication recombination and repair, cell adhesion, metabolism and transport. Each sorafenib-treated HCC cell line displayed specificity in the expression and activity of crucial factors involved in hepatocarcinogenesis.

The p21-activated protein kinase 1 (PAK1) kinase also interacts with the Ras/Raf/MEK/ERK and the PI3K/PTEN/Akt/mTOR cascades. PAK signaling molecules are downstream effectors of Rho family GTPase and interact with both Raf and Akt. PAK inhibitors have been developed [27]. Interestingly, IPA3 inhibits the proliferation of melanoma and colorectal cancer cells with mutations at *KRAS* or *NRAS* better, than those containing mutations at *BRAF*. Treatment of cells with IPA3 or ectopic expression of DN PAK1 sensitized cell with *RAS* mutations to the B-Raf inhibitor GDC-0897 or the MEK inhibitor ZD6244.

Neurofibromin is a GTP-ase activating protein (GAP) which is mutated in neurofibromatosis patients and is considered a tumor suppressor. Neurofibromin normally can regulate cell motility often via the Ras/Raf/MEK/ERK cascade by an interaction of the Ras GTPase-activating protein-related domain (GRD) present on Neurofibromin. Neurofibromin can also regulate Rho-dependent (Ras-independent) event by activating LIM kinase 2 (LIMK2). LIMK2 can phosphorylates and inactivates cofilin (a key protein involved in actin-depolymerization factor). Finally, the pre-GRD domain present on Neurofibromin can interact with Rac1 GTPase, which activate the P21 activated kinase 1 (PAK1)-LIMK1-cofilin pathway. T56-LIMKi is a novel inhibitor that was isolated by molecular modeling that suppressed LIMK1/2 kinase activities which blocked the phosphorylation of cofilin and disrupted actin structure and prevented effects associated with tumorigenicity. The combined effect of the Ras inhibitor Salirasib and T56-LIMKi on cell proliferation were examined and synergistic effects were observed [28].

The PIM kinases can also interact with the EGFR/Ras/Raf/MEK/ERK pathway and certain PIM kinase inhibitors will induce the *MIG6* gene which encodes a suppressor of EGFR signaling. These PIM specific inhibitors (M-110 and SGI-1776) suppress ERK activation in prostate cancer cells. Moreover synergistic effects were observed when the PIM inhibitors were combined with the EGFR inhibitor Gefitinib. These results indicate that the efficacy of EGFR inhibitors may be improved by PIM inhibitors [29].

Ras and other transformed cells often become addicted to autophagy. Targeting certain Ras-addicted cells with autophagy inhibitors (chloroquine and the derivative hydroxychloroquine) may prove to be an effective therapeutic approach [30].

## Rationale for Targeting Multiple Points in Ras/Raf/MEK ERK Pathway or Multiple Pathways

An emerging concept in cancer therapy is the targeting of multiple points in a single signaling pathway. It has been proposed that targeting of both Raf and MEK or Raf and ERK may be useful in certain cancer therapies [31]. The concept of targeting both Raf and MEK is especially true with BRAF inhibitors and melanoma which contain mutations at BRAFV600E due to the negative feedback elicited by downstream ERK which suppresses the sensitivity of the cells to signaling induced by growth factors and Ras activity is low [32]. In these cells the mutant BRAF protein functions as a monomer. The Raf inhibitors suppress the activity of the BRAF monomers but not dimers. Inhibiting the BRAFV600E protein by the BRAF inhibitors inhibits the BRAFV600E protein which suppresses the normal negative ERK-mediated feedback of this pathway. However, this results in reactivation of ligand-dependent signal transduction and increased Ras activity and induces generation of Raf-inhibitor resistant Raf dimers and activation of MEK and downstream ERK. Thus combined Raf and MEK inhibitor treatment may become effective anti-cancer approach. Targeting both MEK and mTOR is a method to target melanoma which often have mutations at *BRAF* and increased activation of the PI3K/PTEN/Akt/mTORC1 pathway [33]. The Ras/Raf/MEK/ERK and PI3K/PTEN/Akt/mTORC1 pathways are regulated by extensive crosstalk, occurring at different levels. In cancer, transactivation of the alternate pathway is a frequent “escape” mechanism. Thus combined inhibition of both pathways may achieve synergistic antitumor activity. In the M14 melanoma model, simultaneous inhibition of both MEK and mTORC1 achieved synergistic effects at suboptimal concentrations.

## Advances in Targeting Anti-Apoptotic Molecules which are Regulated by Ras/Raf/MEK/ERK and PI3K/PTEN/Akt/mTOR Pathways

The effects of combining MEK and Bcl-2 inhibitors on AML cells has been investigated [34]. Anti-apoptotic proteins such as Bcl-2, Bcl-X_L_ and Mcl-1 are key therapeutic targets in human cancer. The mechanisms of actions the Bcl-2 inhibitor ABT-737 have been further elucidated [34]. This Bcl-2 inhibitor suppresses Bcl-2 and Bcl-X_L_ but not Mcl-1. ABT-737 treatment results in activation of the Raf/MEK/ERK cascade and downstream Mcl-1 in AML cells. The MEK inhibitor PD0325901 suppresses Mcl-1 expression. Combining PD0325901 with ABT-737 resulted in synergistic killing of AML-derived cell lines, primary AML blasts and the CD34^+^, CD38^−^, CD123^+^ population which is enriched in progenitor/stem cells. These studies suggest a novel and effective therapeutic strategy for patients with AML.

Treatment of AML cells with ABT-737 and PI3K/mTOR inhibitors (BEZ235 or PI-103) resulted in a synergistic response in AML cells, but importantly not in normal CD34^+^ cells [35]. This synergy was shown to result from inhibition of Bcl-2 and Bcl-X_L_ and the effects of PI3K/mTOR inhibitors were shown to be GSK-3 and Bim dependent. The PI3K/mTOR inhibitors downregulated Mcl-1 but increased Bim binding to Bcl-2/Bcl-XL. Knock-down of GSK-3alpha/beta prevented Mcl-1 downregulation and decreased apoptosis induced by PI3K/mTOR inhibitors. Combining PI3K/Akt/mTOR inhibitors with BH3-mimetics may be an approach to treat AML especially in those patients who exhibit Akt activation [37].

## Advances in Targeting Cancer Initiating Cells

The Ras/Raf/MEK/ERK, PI3K/PTEN/Akt/mTOR, Wnt/beta-catenin, Notch, Hedgehog and other pathways are being shown to play key roles in cancer initiating cells (CICs) and leukemia initiating cells (LIC). Deregulated expression of oncogenes such as Raf-1 have been associated with drug resistance, epithelial to mesenchymal transition and CIC survival [38]. Following metastatic dissemination, cancer cells often re-activate certain epithelial properties through mesenchymal to epithelial transition (MET) to establish neoplastic lesions at secondary sites. The molecular mechanisms regulating MET remain elusive. Recently MET has been examined in estrogen-receptor alpha positive (ERalpha+) MCF-7 breast cancer cells which overexpress activated Raf-1 and are more resistant to the chemotherapeutic drug doxorubicin than parental MCF-7 cells. Constitutive expression of activated Raf-1 induced HER-2/Neu overexpression and lead to distant metastases in xenografts. The development of distant metastases in xenograft models was linked to activation of the MET pathway as the cells expressed reduced expression of EMT inducer genes (*TGFB2*, *TWIST1* and *FOXC1*) and overexpression of *BMB7*, *CXCR7* and early growth response (EGR) family of transcription factors.

The mitotic kinase Aurora-A promotes metastases by inducing EMT transition in ERalpha+ MCF-7 breast cancer cells which expressed constitutively-active Raf-1 [39]. Constitutive expression of activated Raf-1 in MCF-7 cells induced stabilization and accumulation of Aurora-A mitotic kinase. This drove the transition from an epithelial to a highly invasive mesenchymal phenotype. This transition resulted in reduced expression of ERalpha, HER-2/Neu over-expression, and loss of CD24 surface receptor (CD24−/low). Importantly, expression of key EMT markers and upregulation of the stemness gene SOX2 was linked to acquisition of stem cell-like properties. The cells had an increased ability to form mammospheres *in vitro* and tumor self-renewal *in vivo*. Moreover, the aberrant Aurora-A kinase activity induced phosphorylation and nuclear translocation of SMAD5, suggesting a novel interplay between Aurora-A and SMAD5 signaling pathways which may be important in the development of EMT, stemness and ultimately tumor progression. Pharmacologic or molecular inhibition of Aurora-A kinase activity restored a CD24^+^ epithelial phenotype. This phenotype was associated with restored ERalpha expression, down-regulation of HER-2/Neu, inhibition of EMT and impaired self-renewal ability and suppression of metastases. Thus mitotic kinase Aurora-A is a promising therapeutic target to selectively eliminate highly invasive cancer cells.

## Advances in Elucidation of Roles of the PI3K/PTEN/Akt/mTOR Pathway in Oncogenesis

The PI3K pathway has recently been shown to be important in c-Myc expression in Burkitt's lymphomagenesis in germinal center B cells [40]. The PI3K pathway is also an emerging target for mantle cell lymphoma as this cascade is upregulated in this cancer [41]. Disruption of PTEN and p53 activity specifically in the thyroid has recently been shown to result in murine models of anaplastic thyroid carcinomas [42]. These models could be important for the development of approaches to target human thyroid carcinomas. The PI3K pathway is important in many cancer including gliomas often due to aberrant PTEN expression. Recently it was show that reduction of *PIK3CA* or *PIK3RA* expression impeded proliferation, migration, and invasion in glioblastoma multiforme cells [43]. In contrast to PI3K-alpha, PI3K-beta is oncogenic in its WT configuration if it is overexpressed. PI3K-beta acts like an oncogenic mutant of PI3K-alpha [44]. PI3K-delta has been shown to have roles in BCR-ABL-mediated chronic lymphocytic leukemia (CLL) by suppressing BCR signaling [45]. Elevated PI3K signaling has been detected in certain breast cancer subtypes and is believed to drive their abnormal proliferation [46]. Novel lipid phosphatases have been shown to be important in regulation of this pathway. Inositol polyphosphate 4-phosphatase type II (INPP4B), is tumor suppressor in implicated in many cancers such as prostate, breast, and ovarian cancers and also potentially in leukemia [47]. Akt has recently been shown to be an important molecule in regulation of homologous recombination and genetic stability in hereditary and sporadic breast cancers [48].

## Advances in Targeting the PI3K/PTEN/Akt/mTOR Pathway

The PI3K/PTEN/Akt/mTOR pathway is also involved in drug resistance, sensitivity to therapy and metastasis [49-58]. *PIK3CA* mutations may act as driver mutations in certain cancers responsible for metastasis [59]. Novel PI3K-alpha inhibitors have been isolated and they inhibit metastasis [60]. Most PI3K inhibitors are cytostatic rather than cytotoxic and it has been questioned whether treatment with a single PI3K inhibitor will be effective [61].

There have been many recent advances in the development of inhibitors which target this pathway. One of the key developments is in dual PI3K/mTOR inhibitors. Waldenstrom's macroglobulinemia proliferates, in part, in response to aberrant PI3K/Akt activity. The dual PI3K/Akt inhibitor NVP-BEZ235 suppresses the growth of the Waldenstrom's anemia cells as well as has effects on the tumor microenvironment [62].

The PI3K/Akt/mTOR signaling network is activated in acute leukemias of both myelogenous and lymphoid lineage, where it correlates with poor prognosis and enhanced drug-resistance. Treatment of AML and ALL with dual PI3K/mTOR inhibitors has been shown to be more effective than treatment with rapamycin which blocks mTORC1 but not mTORC2 [63]. The dual PI3K/mTOR inhibitors suppressed the rapamycin-resistant phosphorylation of eukaryotic initiation factor 4E-binding protein 1. The novel dual PI3K/mTOR inhibitor NVP-BEZ235, an orally bioavailable imidazoquinoline derivative, has entered clinical trials. Moreover in both T-ALL cell lines and patient samples. NVP-BEZ235 was cytotoxic to a panel of T-ALL cell lines as determined by MTT assays. NVP-BEZ235 induced cell cycle arrest and apoptosis. A dose- and time-dependent dephosphorylation of Akt and mTORC1 downstream targets was observed after NVP-BEZ235 treatment. NVP-BEZ235 targeted the side population of both T-ALL cell lines and patient lymphoblasts, which is enriched in leukemia initiating cells (LIC), and synergized with chemotherapeutic agents (cyclophosphamide, cytarabine, dexamethasone) which are used currently for treating T-ALL patients. NVP-BEZ235 reduced chemoresistance to vincristine induced in Jurkat T cells upon coculturing with MS-5 stromal cells, which mimics the bone marrow microenvironment. NVP-BEZ235 was cytotoxic to T-ALL patient lymphoblasts displaying pathway activation, where the drug dephosphorylated eukaryotic initiation factor 4E-binding protein 1, in contrast rapamycin did not elicit such changes. Longitudinal inhibition at two nodes of the PI3K/Akt/mTOR network with NVP-BEZ235, either alone or in combination with chemotherapeutic drugs, may be an efficient treatment of those T-ALLs that have aberrant upregulation of this signaling pathway for their proliferation and survival [64].

The dual PI3K/mTOR inhibitor NVP-BEZ235 has also been shown recently to synergize with a pan-histone deacetylase inhibitor in suppressing the growth of pancreatic cancer [65].

Effective Akt inhibitors have been recently developed. The Akt inhibitor MK-2206 has been shown to be effective in suppressing the growth of many cancers including leukemias. This Akt inhibitor is currently in many clinical trials with cancer patients have diverse diseases either as a single or combined agent with an additional signal transduction inhibitor or chemo- or endocrine therapy [65]. The PI3K/PTEN/Akt/mTORC1 pathway is frequently upregulated in T-ALL, The effects of the novel allosteric Akt inhibitor, MK-2206, on a panel of human T-ALL cell lines and primary cells from T-ALL patients were examined. MK-2206 decreased cell viability by blocking leukemic cells in G_0_/G_1_ and induced apoptosis as well as autophagy and a concentration-dependent dephosphorylation of Akt and its downstream targets, GSK-3alpha/beta and FOXO3A. MK-2206 was cytotoxic to primary T-ALL cells and importantly induced apoptosis in the T-ALL patient cell subset (CD34^+^, CD4^−^, CD7^−^), which are enriched in LICs. Akt inhibition may represent a potential therapeutic strategy in T-ALL.

Alkylphospholipids and alkylphosphocholines (APCs) are promising antitumor agents, which target the plasma membrane and affect multiple signal transduction networks including Akt. The therapeutic potential of erucylphosphohomocholine (ErPC3), the first intravenously applicable APC, on human AML cells was determined. At short (6-12 h) incubation times, ErPC3 blocked cells in G_2_/M phase of the cell cycle, whereas, at longer incubation times, it decreased survival and induced apoptotic cell death. ErPC3 induced JNK 1/2 activation while stimulating ERK 1/2 dephosphorylation. Pharmacological inhibition of caspase-3 or a JNK 1/2 inhibitor peptide reduced ErPC3 cytotoxicity. Protein phosphatase 2A downregulation by siRNA inhibited ERK 1/2 dephosphorylation and blunted the cytotoxic effects of ErPC3. ErPC3 was also cytotoxic to AML primary cells and importantly reduced the clonogenic activity of CD34^+^ leukemic cells. ErPC3 treatment induced apoptosis in the (CD34^+^, CD38(Low/−), CD123^+^) compartment which is enriched in putative LICs. ErPC3 induced cytotoxicity on AML blasts which expressed high levels of aldehyde dehydrogenase activity and on the side population of AML cell lines and blasts in which the LIC populations are believed to reside. Thus ErPC3 might be a promising therapeutic agent for the treatment of AML patients [66].

## Advances in Understanding the Pleiotropic Effects of mTOR

The importance of mTOR and its inhibition in various conditions, including cancer, diabetes, aging and others has been further elucidated. Recently mTOR has been shown to be cell cycle regulated [67,68]. mTOR has been referred to as the gatekeeper of autophagy. mTOR plays important roles in many biological processes, including; autophagy [69] energy control [70-72], insulin resistance [73], diabetes [74,75], seizures [76,77], protein homeostasis [78], regulation of tRNA expression [79,80], cell cycle arrest [81], cell differentiation [82,83], cell migration [84,85], follicle development [86], DNA damage checkpoint [87], DNA replication stress [88],cellular quiescence/senescence [89-107] progeria [108], age-related retinal diseases [109], obesity [110], stem cells [111], cancer [112,113], aging [114-134], Alzheimer's disease [135] and Parkinson's disease [136].

mTORC1 is a critical repressor of autophagy, a lysosome-dependent degradation pathway which allows cells to recycle damaged or unnecessary cytoplasmic content, such as lipids, proteins, and organelles [138-156]. As a consequence, cells produce metabolic precursors for macromolecular biosynthesis or ATP generation. In cancer cells, autophagy fulfills a dual role, as it has both tumor-promoting and tumor-suppressing properties. Functional autophagy prevents necrosis and inflammation, which can lead to genetic instability. However, autophagy is important for tumor progression by providing energy through its recycling mechanism during unfavorable metabolic circumstances, which frequently occurs in certain tumors [138-156].

A model was proposed recently by Dr. Michael P. Lisanti and colleagues which is called the reverse Warburg Effect. This model proposes that the aerobic glycolysis occurring in the tumor associated fibroblasts and not in the actual epithelial tumor cells [142,146-150]. This results in the transfer of high-energy metabolites (lactate and pyruvate) to adjacent epithelial cancer cells which fuel the cancer cells allowing them to invade and metastasize. In addition, oxidative stress generated by the cancer cells induces autophagy of the tumor associated fibroblasts which the cancer cells then recycle and use to fuel their growth. Anti-oxidants (N-acetyl cysteine, NAC), quercetin and the anti-diabetes drug metformin) or autophagy inhibitors (chloroquine) will suppress the destruction of caveolin-1 in stromal fibroblasts and inhibit cancer growth. Caveolin-1 is a key protein at the cell membrane which serves to organize other important signaling molecules into signaling complexes (*e.g*., Fak, Src). Decreased expression of caveolin-1 is associated with a poorer prognosis of breast and other cancers.

Autophagy is also important in blood cancers [151-153]. Autophagy can be regulated by epigenetic mechanisms [154]. Autophagy may also become defective in certain drug resistant cells [155]. Defective autophagy may be controlled by the p53 rheostat in cancer [156]. Clearly autophagy is a very important survival process which is regulated in part by mTORC1.

The mTORC1 blocker rapamycin may be useful in the treatment of many diseases including HIV and HCV infections [157,158].

## Advances in mTOR Kinase Inhibitors

mTOR kinase inhibitors have been developed and evaluated on many diseases including leukemias [159]. mTOR is present in two complexes, mTORC1 and mTORC2. Both of these complexes play critical roles in signal transduction pathways and regulate cell growth and many other physiological processes. Rapamycin and rapalogs only inhibit (block) mTORC1. Allosteric inhibition of mTORC1 by rapamycin had only modest effects in T-ALL. ATP-competitive inhibitors specific for the mTOR kinase active site have been developed recently. The therapeutic potential of active-site mTOR inhibitors in suppressing growth in T-ALL cell lines and primary samples from T-ALL patients which had activation of mTORC1 and mTORC2 was examined. The inhibitors affected T-ALL cell viability by inducing G_0_/G_1_ cell cycle arrest, apoptosis and autophagy. Decreased levels of the mTORC2 target Ser 473 Akt were detected as well as dephosphorylation of mTORC1 downstream targets. Unlike rapamycin, marked inhibition of mRNA translation in T-ALL cell lines treated was observed with active-site mTOR inhibitors. The mTOR inhibitors synergized with both vincristine and the Bcl-2 inhibitor, ABT-263. The mTOR inhibitors targeted a putative LIC sub-population (CD34^+^, CD7^−^, CD4^−^) in the ALL patient samples. The mTOR inhibitors displayed remarkable anti-leukemic activity, and could become clinical candidates for T-ALL therapy [160].

## Advances in Targeting the NF-kappaB Pathway

Targeting the NF-kappaB pathway has been shown to be a potential therapeutic approach in leukemia and other therapies. The NF-kappaB pathway is regulated in part by PI3K/PTEN/Akt/mTOR pathway. The IkappaB kinase (IKK)/NF-kappaB axis is required for viability of leukemic cells and is a predictor of relapse in T-ALL. It turns out that many anticancer agents induce NF-kappaB nuclear translocation. This results in activation of NF-kappaB target genes, which can alter the sensitivity to chemotherapeutic drugs. The I-kappaB kinase inhibitor is the target of BMS-345541 [161]. The anti-proliferative effects of BMS-345541 in three Notch1-mutated T-ALL cell lines and in T-ALL primary cells from pediatric patients were investigated. BMS-345541 induced apoptosis and accumulation of cells in the G _2_/M phase of the cell cycle. T-ALL cells treated with BMS-345541 displayed nuclear translocation of FOXO3a as well as downstream p21^Cip1^ expression. FOXO3a subcellular re-distribution was independent of AKT and ERK 1/2 signaling, suggesting that in T-ALL, the loss of FOXO3a tumor suppressor function could be due to deregulation of IKK. FOXO3a mutations are not frequently found in human tumors. Thus therapeutics activating FOXO3a may be more effective than others. BMS-345541 could be used alone or in combination with traditional therapies in the treatment of T-ALL.

Dehydroxymethyl-epoxyquinomicin (DHMEQ) is an inhibitor of NF-kappaB [162] DHMEQ induces apoptosis through reactive oxygen species (ROS) production in hepatoma cells. Cox-2 inhibition is an additional approach to inhibit hepatoma cell growth [163]. Celecoxib can also synergize with the proteasome inhibitor MG132 and suppress the growth of hepatocellular carcinoma [164]. Subsequently DHMEQ was determined to synergize with the Cox-2 inhibitor celecoxib in hepatoma cells, again via a ROS-dependent mechanism [165]. DHMEQ cooperated with celecoxib (CLX) to decrease NF-kappaB DNA binding and inhibited cell proliferation more effectively than treatment with these single agents alone. DHMEQ-CLX combination resulted in ROS production which in turn induced the expression of genes involved in endoplasmic reticulum (ER) stress. Silencing TRB3 mRNA, which is associated with ER stress, significantly decreased DHMEQ-CLX-induced cell growth inhibition. The DHMEQ-CLX combined treatment was associated with induction of PARP cleavage and down-regulation of the anti-apoptotic proteins Bcl-2, Mcl-1 and survivin, as well as activated Akt. CD95 and CD95 ligand expression increased synergistically in the combined treatment, which was reversed in the presence of NAC. Knockdown of CD95 mRNA expression decreased DHMEQ-CLX-induced growth inhibition in HCC cell lines. Thus the DHMEQ-CLX combination killed hepatoma cells via ROS production, ER stress response and the activation of intrinsic and extrinsic apoptotic pathways.

## Advances in Targeting Multiple Points in PI3K/PTEN/Akt/mTOR pathway

An emerging concept in cancer therapy is the targeting of multiple points in the PI3K/PTEN/Akt/mTOR and other pathways. The expression of the PI3K/PTEN/Akt/mTOR pathway is often upregulated in melanomas. The dual PI3K/mTOR inhibitor NVP-BEZ235 inhibited melanoma growth regardless of *BRAF* mutation status. Rapamycin enhanced the activity of the dual PI3K/mTOR inhibitor NVP-BEZ235 in inhibiting the growth of melanoma [166]. Furthermore addition of the MEK inhibitor AZD6244 synergized with the dual PI3K/mTOR inhibitor NVP-BEZ235 in suppressing melanoma growth.

The effects of targeting different levels of the PI3K/PTEN/Akt/mTOR pathway has also been examined in various leukemias [167]. The efficacy of co-targeting of different components of the PI3K/PTEN/Akt/mTOR pathway with a novel dual PI3K/Akt inhibitor has been investigated in T-ALL. [168]. The dual PI3K/PDK1 inhibitor NVP-BAG956 was determined in one study to exert the most powerful cytotoxic affects against T-ALL cell lines and primary patients samples, in comparison to a pan class I PI3K inhibitor (GDC-0941), an allosteric Akt inhibitor (MK-2206), an mTORC1 allosteric inhibitor (RAD-001), or an ATP-competitive mTORC1/mTORC2 inhibitor (KU63794).

Often signal transduction inhibitors are cytostatic as opposed to cytotoxic. This is especially true with rapamycin. The effects of combining targeted therapy with chemotherapy is an emerging concept and is also being examined by clinical trials [169]. The therapeutic potential of a combination of temsirolimus [an allosteric mTOR complex 1 (mTORC^1^) inhibitor] with clofarabine, a nucleoside analogue with potent inhibitory effects on both ribonucleotide reductase and DNA polymerase was examined in AML cell lines and clinical specimens. The drug combination (CLO-TOR) displayed synergistic cytotoxic effects against a panel of AML cell lines and in primary cells from AML patients. The CLO-TOR treatment induced arrest at the G0/G1 phase of the cell cycle, apoptosis, and autophagy. CLO-TOR combination was pro-apoptotic in an AML patient blast subset (CD34^+^, CD38^−^, CD123^+^), which is enriched in putative LICs. The CLO-TOR combination could represent a novel valuable treatment for AML patients, also in light of its efficacy against LICs.

## Activation of Liver Kinase B1/AMP Activated Protein Kinase Pathway as an Anti-Cancer Therapy

The liver kinase B1/AMP activated protein kinase (LMPK) pathway has been determined to be a key pathway in metabolism (diabetes) as well as cancer and other diseases [170]. The LKB1/AMPK network remains functional in a wide range of cancers and can be stimulated by drugs, such as N,N-dimethylimidodicarbonimidic diamide (metformin) or 5-aminoimidazole-4-carboxamide 1-β-D-ribofuranoside (AICAR). LKB1/AMPK signaling induces cell cycle arrest, caspase-dependent apoptosis or autophagy in various tumors. Metformin inhibits mTORC1-controlled oncogenetic protein translation, which does not occur with allosteric mTORC1 inhibitors, such as rapamycin and its derivatives. Metformin also targets LICs and CICs, the critical target for leukemia eradication. Thus the LKB1/AMPK pathway is critically involved in regulating proliferation and survival of malignant cells. Drugs activating LKB1/AMPK may be both a novel and less toxic treatment option for certain malignancies [171-174].

The effects of metformin against T-ALL cell lines and primary samples from T-ALL patients displaying mTORC1 activation. Metformin inhibited T-ALL cell viability by inducing autophagy and apoptosis. Importantly, it was much less toxic against proliferating CD4^+^ T-lymphocytes from healthy donors. Dephosphorylation of downstream targets of mTORC1 were detected. A marked inhibition of mRNA translation in T-ALL cells treated with metformin, in contrast such an inhibition of translation was not observed after rapamycin treated. Remarkably, metformin targeted the side population of T-ALL cell lines as well as a putative LIC subpopulation (CD34^+^, CD7^−^, CD4^−^) in primary patient samples. Metformin displayed a remarkable anti-leukemic activity, which emphasizes future development of LKB1/AMPK activators as clinical candidates for therapy of T-ALL [175]. AMPK has also been shown to be important in BCR-ABL-induced CML and AMPK activators may prove useful as supplemental drugs for this disease [176]. AMPK can also be activated by rapamycin [177].

The plant natural product berberine has recently been shown to inhibit the growth of drug resistant breast cancer cells whereas the parental drug sensitive line was not as growth inhibited [178]. Berberine may also target LKB1/AMPK. Breast cancer cells overexpressing neutrophil gelatinase associated lipocalin (NGAL) were more sensitive to berberine than parental cells which did not over express NGAL [178].

## Advances in Understanding the p53 Pathway and Targeting

Both the PI3K/PTEN/Akt/mTOR and Ras/Raf/MEK/ERK pathways can interact with the p53 pathway at various levels. Functional p53 gene status was determined to be important in the sensitivity of prostate cancer cells to chemotherapeutic drugs, radiation treatment and the small molecule MDM2 inhibitor Nutlin 3A. [179]. The p53 transcription factor is a critical element in the ability of the cell to regulate the cell cycle and its response to DNA damage. Mutations within the DNA-binding domain of p53 are common in human cancers and allow the formation of tetramers; however, these alterations prevent this protein complex from associating with appropriate target gene promoters. The effects of p53 functionality in prostate cancer cells that harbored WT or mutant forms of the protein in response to commonly used chemotherapeutic drugs were examined. The androgen receptor positive 22Rv-1 and LNCaP prostate cancer cell lines carry WT p53 and were demonstrated to have a decrease in chemotherapeutic drug sensitivity when transfected with a dominant-negative (DN) p53 gene. Conversely, expression of a WT p53 gene in the p53-mutated and more advanced DU145 prostate cancer cell line significantly increased its overall sensitivity to anti-neoplastic drugs. Analysis of colony formation in soft agar revealed that the functional status of p53 in each cell line altered the ability to proliferate in an anchorage-independent fashion. Prostate cancer colony growth was more prevalent when p53 transcriptional activity was decreased, whereas growth was more limited in the presence of functional p53. Thus the functional status of the tumor suppressor p53 is important in the progression of prostate cancer and dictates the overall effectiveness a given drug would have on disease treatment.

p53 has also been shown to be a target for lowering associated toxicity of normal cells after chemotherapy. The protection of normal cells from cell cycle-specific chemotherapeutic agents such as mitotic inhibitors (MI) was examined after a 3-day exposure to MI (paclitaxel and nocodazole) by colony formation. In three normal human cell types with WT-p53 (RPE, NKE, WI-38t cells) but not in cancer cells with mutant p53, pre-treatment with Nutlin-3a, caused G1 and/or G2 arrest, thus preventing lethal mitotic arrest normally induced by MIs and allowing normal cells to recover after removal of the MIs. Rapamycin potentiated the protective effects of Nutlin-3a in the p53-WT cells. Also, a combination of rapamycin and metformin, induced G_1_ and G_2_ arrest selectively in p53-WT cells and thereby protected them from the MIs. A combination of metformin and rapamycin also protected p53-WT cells in low glucose conditions, whereas it was cytotoxic for p53 mutant cells. A rational combination of metformin and rapamycin may potentiate chemotherapy with MIs, while protecting normal cells thus therapy-induced toxicities [180-181].

## Advances in Roles of Androgen Receptor, Beta-Catenin, and Akt in Androgen Responsiveness of Prostate Cancer

The effects of silencing androgen receptor (AR), beta-catenin and Akt expression in prostate cancer growth and migration were examined [182]. The mechanisms responsible for the conversion of prostate cancer from androgen-sensitive (AS) to androgen-insensitive (AI) are not well understood. AR signaling involves cross-talk with the other signaling pathways, and other proteins, such as beta-catenin. To further elucidate some of the biochemical changes that occur during the switch from AS to AI form, AR, Akt and β-catenin expression were knocked-down with respective siRNAs. Treatment of AR+ LNCaP prostate cells with siRNA for AR significantly reduced proliferation (45-70%), expression of nuclear β-catenin, cyclin-D1, cyclin-G1, c-Myc as well as activity of metalloproteinases (MMPs) −2,−7,−9 and cell migration. After longer periods of AR silencing (over 72 hrs) in LNCaP cells, elevated levels of activated Akt were detected and enhanced proliferation as well as expression of nuclear beta-catenin, cyclin-D1, c-Myc and activity of MMPs were observed. Such effects were not observed in either AI, AR-, PC-3 or DU145 cells. However, silencing of Akt and /or beta-catenin in those as well as in AS, AR+ LNCaP cells led to decreased proliferation and migration. These studies indicate that either AR or Akt signaling prevails, depending on their initial androgen sensitivity as well as its availability. In AI prostate cancer cells, Akt takes over the role of AR and more effectively contributes through the same signaling molecule, beta-catenin, to AI cancer progression. Thus Akt can be a key target in prostate cancer.

## Advances in NO-Modified Drugs and Inhibitors

Recently the effect on nitric oxide (NO)-modified inhibitors and drugs has been examined in various cancer settings [183-185]. The NO-modified form of HIV protease inhibitor Saquinavir (Saq) is a potent antitumoral agent efficient against numerous tumor cell lines in vitro and in vivo. Saq-NO was determined to sensitize certain types of cells to tumor necrosis factor-related apoptosis-inducing ligand (TRAIL)-mediated cell death. Saq-NO inhibited both the growth of LNCaP cells in vitro and in xenograft models. Suppression of tumor growth was accompanied with cell cycle arrest in G_0_/G_1_ phase. Permanent abrogation of S6 phosphorylation was observed. Diminished S6 phosphorylation was associated with re-established sensitivity to TRAIL and reduction of X-linked inhibitor of apoptosis protein (XIAP). NO modification of Saq led to a new chemical entity with stronger and more pleiotropic antitumor activity than the parental drug.

The effects of the NO-modified anti-inflammatory drug (S,R)-3-phenyl-4,5-dihydro-5-isoxasole acetic acid (VGX-1027) named GIT-27NO or the NO-modified antiviral drug saquinavir (Saq) named Saq-NO were examined on colon cancer cell lines, murine CT26CL25 and human HCT116. Both agents suppressed the growth of colon cancer cells in vitro. The efficacy of the drugs was evaluated in vivo in BALB/c mice injected with CT26CL25 cells. Both agents suppressed reduced tumor volume in syngeneic BALB/c mice. However, their mechanisms of action were different as GIT-27NO released larger amounts of nitrite than Saq-NO in vitro and its antitumor action depended on the intracellular NO release. In contrast, Saq-NO released barely detectable amounts of NO and its antitumor action was NO-independent. Cotreatment with an NO-peroxynitrite scavenger revealed that GIT-27NO but not Saq-NO acts through peroxynitrite-mediated cell destruction. GIT-27NO predominately induced proapoptotic signals followed by caspase-dependent apoptosis. While Saq-NO blocked cell proliferation, changed their adhesive, migratory, and invasive properties, and decreased their metastatic potential in vivo. In conclusion, differences in NO release and oxidative stress generation between GIT-27NO and Saq-NO resulted in different mechanisms that resulted in cell death [186].

## Advances in Elucidating Role of NGAL in Cancer

Neutrophil gelatinase associated lipocalin (NGAL aka lipocalin-2 or siderocalin) has been shown to play diverse roles, from stabilizing matrix matalloproteinase (MMP-9) to combating bacterial infection by binding bacterial siderophores and preventing bacterial iron sequestration to roles in cancer invasion, EMT, and metastasis [187]. NGAL is believed to play roles in promoting survival, growth, invasion and metastasis. In addition NGAL may also have roles in sequestration of iron resulting in cell survival and tumorigeneis. Upregulated NGAL expression in often upregulated in advanced tumors and is easily detected in urine. Clearly NGAL may represent an important marker for certain cancers.

Recently the role of NGAL in sensitivity to targeted therapy has been investigated in breast cancer cells. Ecotopic expression of NGAL does not increase the resistance of cancer cells to doxorubicin [188]. In contrast, ectopic expression of NGAL did alter the sensitivity of MCF-7 breast cancer cells to targeted therapy [178] MCF-7/NGAL were more sensitive to EGFR, Bcl-2 and calmodulin kinase inhibitors as well as the natural plant product berberine than MCF-7 cells infected with the empty retroviral vector pLXSN. These results are important as the expression of NGAL is often detected at elevated levels after chemotherapy [189]. Furthermore the expression of NGAL increases in more advanced cancers.

## SUMMARY

In this review, we have discussed some of the recent advances in targeting certain signal transduction pathways. Although there have been many advances in our understanding of other key pathways involved in cancer such as Wnt/beta-catenin [190], Notch [191] and hedgehog [192], we have primarily focused on the Ras/Raf/MEK/ERK and PI3K/PTEN/Akt/mTOR pathways due to space considerations. Clearly as we learn more about these pathways, we find that they have not only complex interactions with other pathways, but the importance of genetics and biochemistry in the sensitivity and resistance to targeted therapy. Further elucidation of these and other signaling pathways may allow more effective therapies to be developed for cancer and other diseases.
